# Supplementary material for: Modeling medulloblastoma in vivo and with human cerebellar organoids
Source: Nat Commun. 2020 Jan 29;11:583. doi: 10.1038/s41467-019-13989-3 (PMC6989674; doi:10.1038/s41467-019-13989-3)
Supplement: Supplementary file 1 — Supplementary Information [file 41467_2019_13989_MOESM1_ESM.pdf]

# Modeling Medulloblastoma *in-vivo* and with human cerebellar organoids

**Ballabio et al.**

A

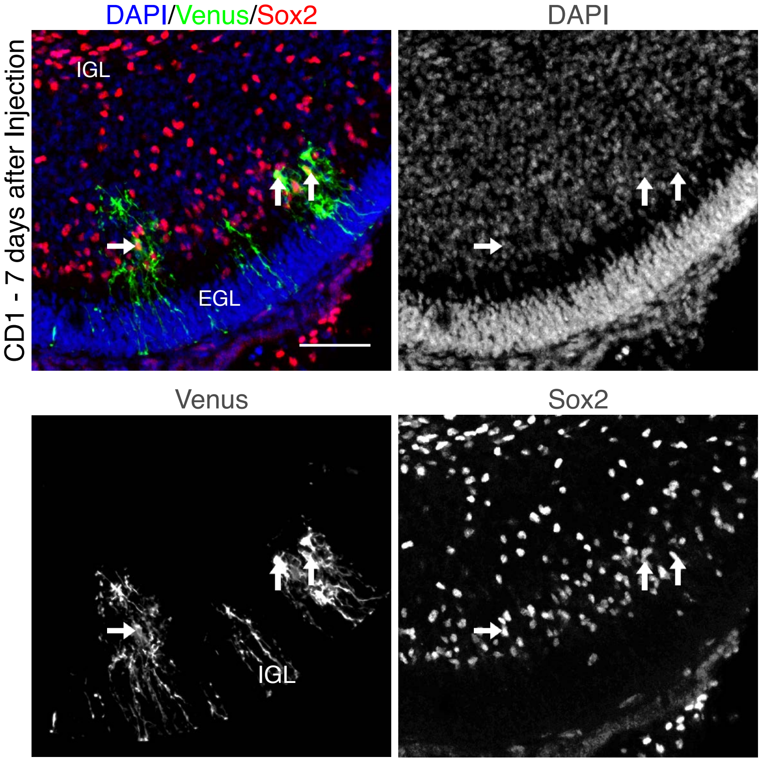


B

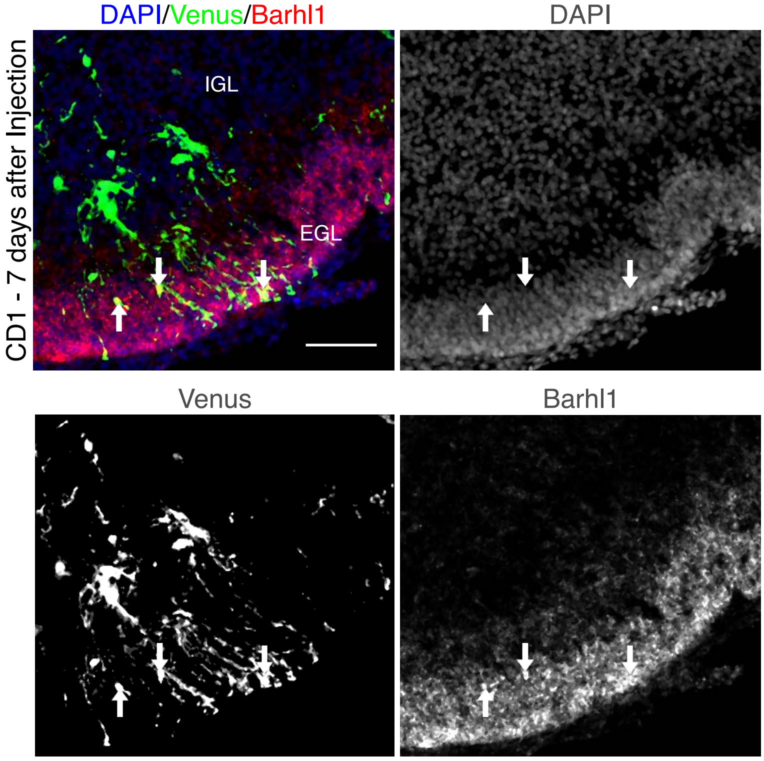


C

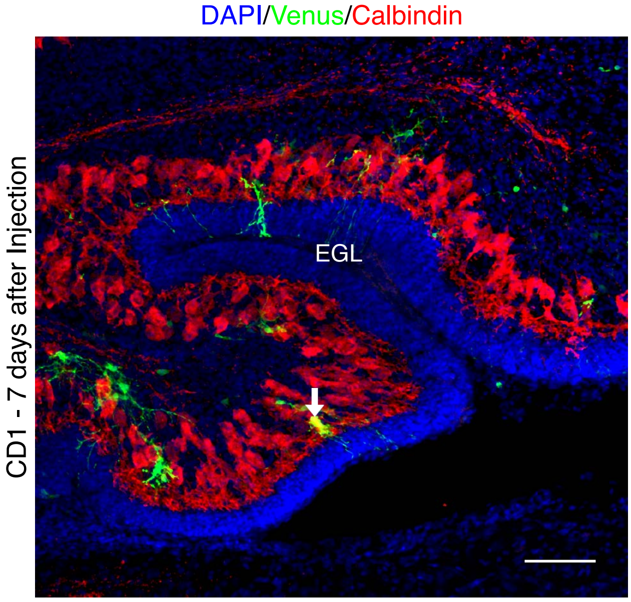


D

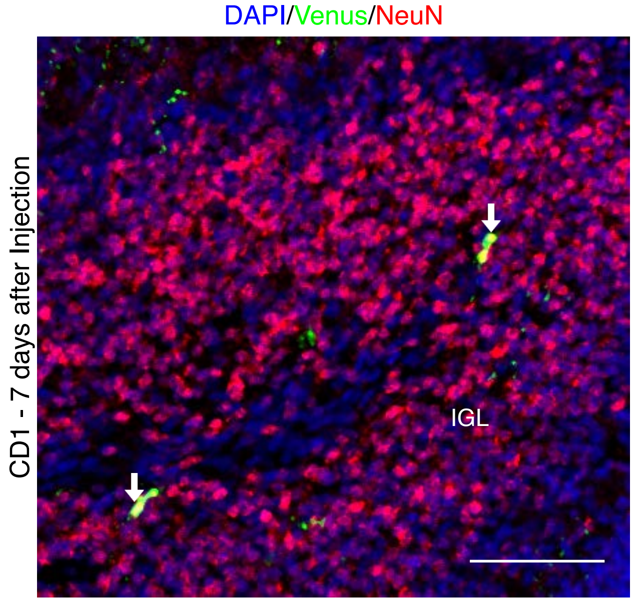


E

| *In vivo* transfection mix | Patients ID |
|---|---|
| Smarca4, Kmt2d, Kmt2c | ICGC_MB226, MD-143 (no KMT2C mutation) |
| Smarca4, Chd7 | MD-284 |
| Gfi1, c-Myc | ICGC_MB18, ICGC_MB50, ICGC_MB304 |
| Gfi1, Smarca4 | |
| Gfi1, Kmt2d | |
| Gfi1, Cdk6 R31C | ICGC_MB57, MDT-AP-0220 |
| Smarca4 T910M,Ctdnep1,Kmt2d | ICGC_MB226 |
| c-Myc, Smarca4 T910M | ICGC_MB226 |
| c-Myc, Smarca4 | ICGC_MB226, ICGC_MB277, ICGC_MB39, ICGC_MB9, MDT-AP-1166 |
| c-Myc, Ctdnep1 | ICGC_MB106, ICGC_MB225, ICGC_MB226, ICGC_MB247 |
| Smarca4, Kbtbd4 | ICGC_MB39, MDT-AP-1166, MDT-AP-2452 |
| Smarca4 T910M, Kbtbd4 | |
| c-Myc, Kmt2d, Kmt2c | ICGC_MB226, ICGC_MB289, SJMB030, MDT-AP-1364 |
| Lrp1b, Kmt2d, Smarca4 | MD-143 |
| Gfi1, c-Myc, Smarca4 WT | ICGC_MB18, ICGC_MB50, ICGC_MB304 |
| Gfi1, Otx2 | ICGC_MB18, ICGC_MB50, ICGC_MB304 |
| Otx2, c-Myc | ICGC_MB106, ICGC_MB111, ICGC_MB131, ICGC_MB134, ICGC_MB139, ... |
| Gfi1, Smarca4 T910M | |
| Otx2, Ctdnep1 | ICGC_MB106, ICGC_MB225, ICGC_MB226, ICGC_MB247 |
| Ddx3x, Kmt2d | |
| Otx2, c-Myc, Smarca4 WT | |
| Otx2 | |
| c-Myc | |
| Gfi1 | |
| Venus | |

Cas9-mediated KO
PiggyBac-mediated overexpression

F

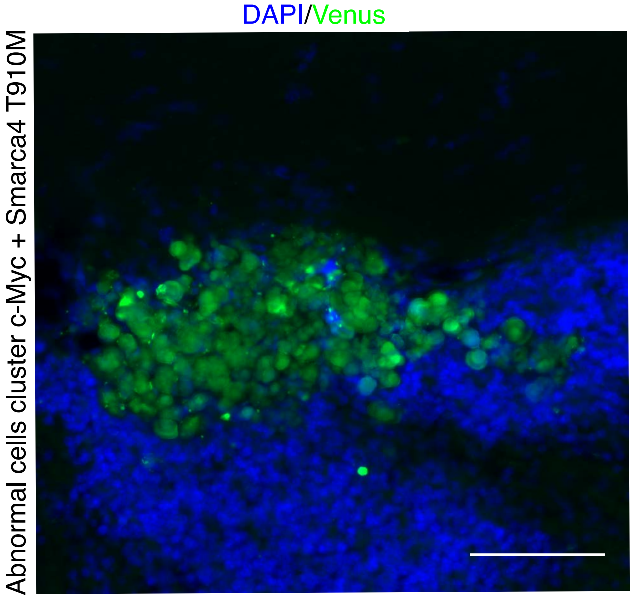


# Supplementary Figure 1

**Supplementary Figure 1 | *In-vivo* transfection of mouse cerebellum**

(**A**) Confocal image of DAPI staining and immunofluorescence for GFP (Venus) and Sox2 of sagittal brain section of CD1 mouse 7 days after transfection with pPBase and pPBVenus at P0. (**B**) Confocal image of DAPI staining and immunofluorescence for GFP (Venus) and Barhl1 of sagittal brain section of CD1 mouse 7 days after transfection with pPBase and pPBVenus at P0. (**C**) Confocal image of DAPI staining and immunofluorescence for GFP (Venus) and Calbindin of sagittal brain section of CD1 mouse 7 days after transfection with pPBase and pPBVenus at P0. (**D**) Confocal images of DAPI staining and immunofluorescence for GFP (Venus) and NeuN of sagittal brain section of CD1 mouse 7 days after transfection with pPBase and pPBVenus at P0. Arrows in (**A,B,C,D**) indicate double positive cells. (**E**) List of combinations of putative oncogenes and putative oncosuppressors transfected in CD1 mice at P0. Patients ID bearing the alterations corresponding to the transfected combinations are reported. (**F**) DAPI staining and immunofluorescence for GFP (Venus) of sagittal brain section of CD1 mouse 3 months after transfection with pPBase and pPBVenus + pPBMyc + pPBSmarca4-T910M + pPBVenus at P0. Scale bars 100 μm in (**A-D,F**).

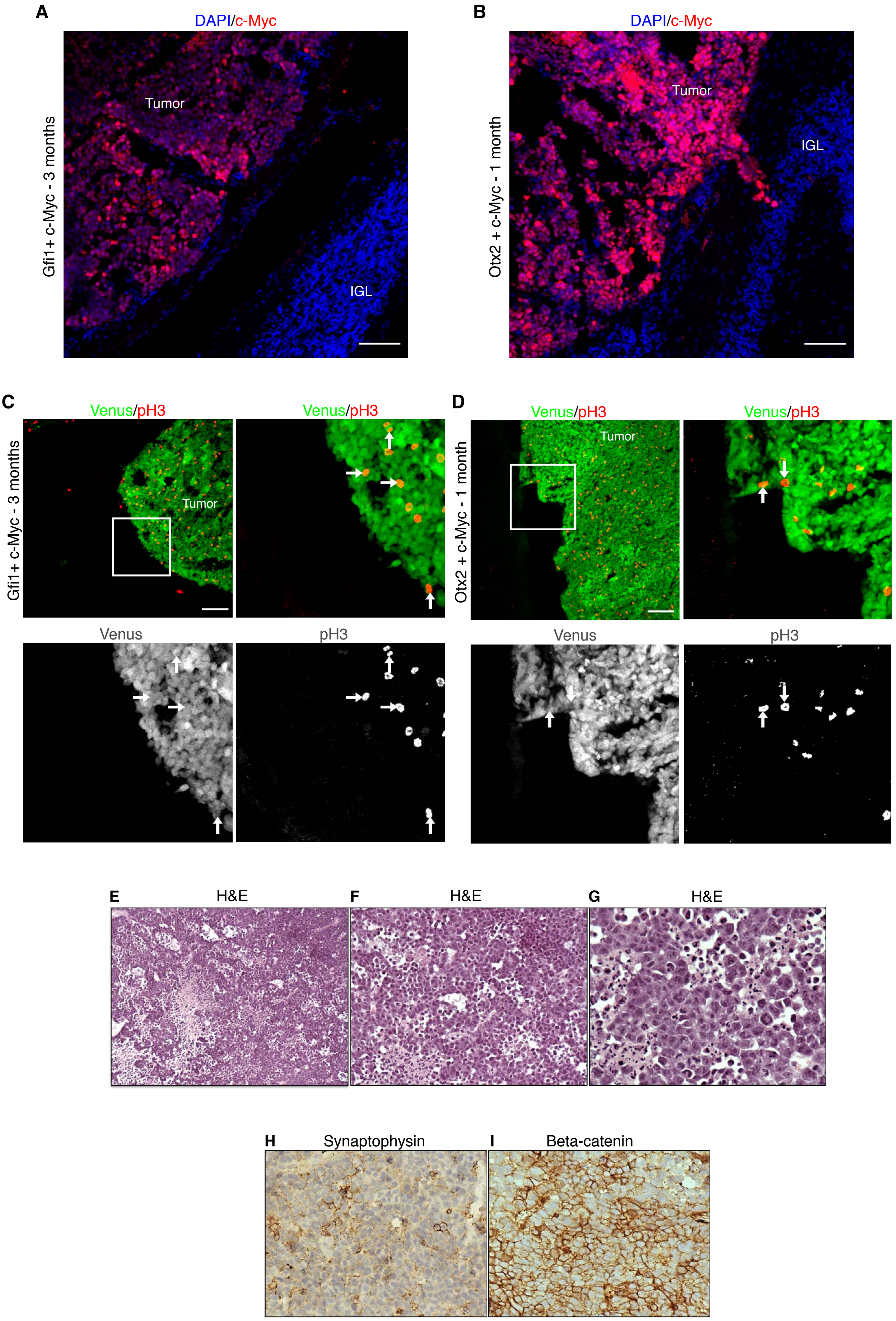


**Supplementary Figure 2**

**Supplementary Figure 2 | c-Myc, Gfi1 and Otx2 expression in mouse MB**

(**A**) Confocal image of DAPI staining and c-Myc immunofluorescence of sagittal brain section of CD1 mouse 3 months after transfection with pPBase + pPBMyc + pPBGfi1 + pPBVenus at P0. (**B**) Confocal image DAPI staining and c-Myc immunofluorescence of sagittal brain section of CD1 mouse 1 month after transfection with pPBase + pPBMyc + pPBOtx2 + pPBVenus at P0. (**C-D**) Confocal images of GFP (Venus) and pH3 immunofluorescence of tumors in CD1 mice after transfection with pPBase + pPBMyc + pPBGfi1 + pPBVenus at P0 (**C**) and with pPBase + pPBMyc + pPBOtx2 + pPBVenus at P0 (**D**). Arrows point to double positive cells. The white square marks the region shown at higher magnification. (**E-G**) H&E stained section. Medulloblastoma at different magnification. (**E)** The tumor is characterized by cells with hyperchromatic nuclei and foci of necrosis (10X magnification). (**F**) Higher magnification showing primitive-appearance cells with high N:C ratio (20X magnification). (**G)** Presence of pleomorphic cells with nuclear molding and many apoptotic cells. Mitotic figures are visible in this image (40X magnification). (**H )** Positive staining for Synaptophysin in the cytoplasm of cells (20X magnification). (**I**) Beta-catenin immunoreactivity restricted to the plasma membrane and cytoplasm identify non-WNT medulloblastoma (20X magnification). Scale bars 100 μm in (**A-D**).

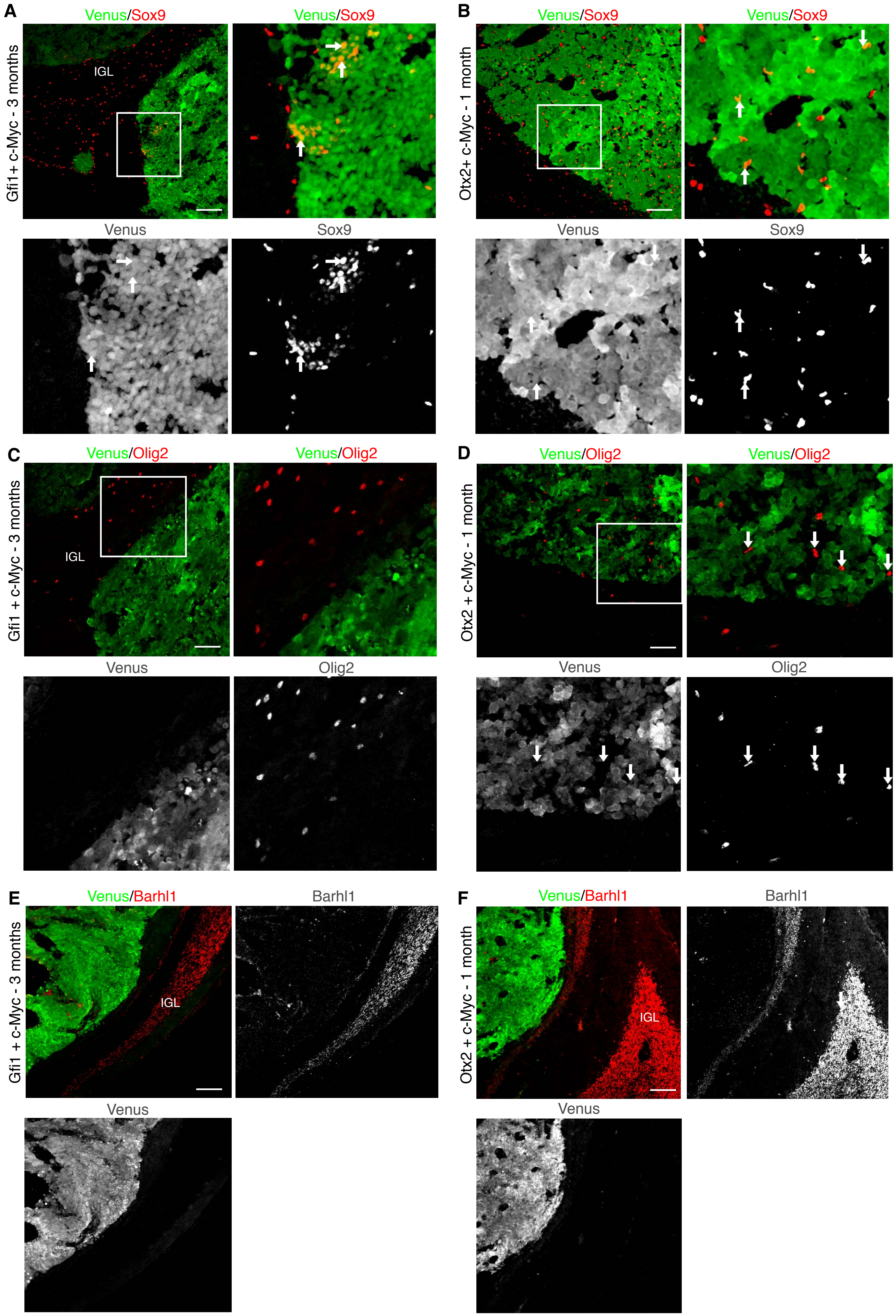


**Supplementary Figure 3**

**Supplementary Figure 3 | *In-vivo* transfection of cerebellar cells with Gfi1/c-Myc and Otx2/c-Myc induces MB**
(**A,B**) Confocal images of GFP (Venus) and Sox9 immunofluorescence of tumors in CD1 mice after transfection with pPBase + pPBMyc + pPBGfi1 + pPBVenus at P0 (**A**) and with pPBase + pPBMyc + pPBOtx2 + pPBVenus at P0 (**B**). Arrows point to double positive cells. (**C,D**) Confocal images of GFP (Venus) and Olig2 immunofluorescence of tumors in CD1 mice after transfection with pPBase + pPBMyc + pPBGfi1 + pPBVenus at P0 (**C**) and with pPBase + pPBMyc + pPBOtx2 + pPBVenus at P0 ( **D**). Arrows point to infiltrated Olig2 positive cells. The white square in (**A,B,C,D**) marks the region shown at higher magnification ( **E,F**) Confocal images of GFP (Venus) and Barhl1 immunofluorescence of tumors in CD1 mouse after transfection with pPBase + pPBMyc + pPBGfi1 + pPBVenus at P0 (**E**) and with pPBase + pPBMyc + pPBOtx2 + pPBVenus at P0 (**F**). Scale bars 100 µm in (**A-F**).

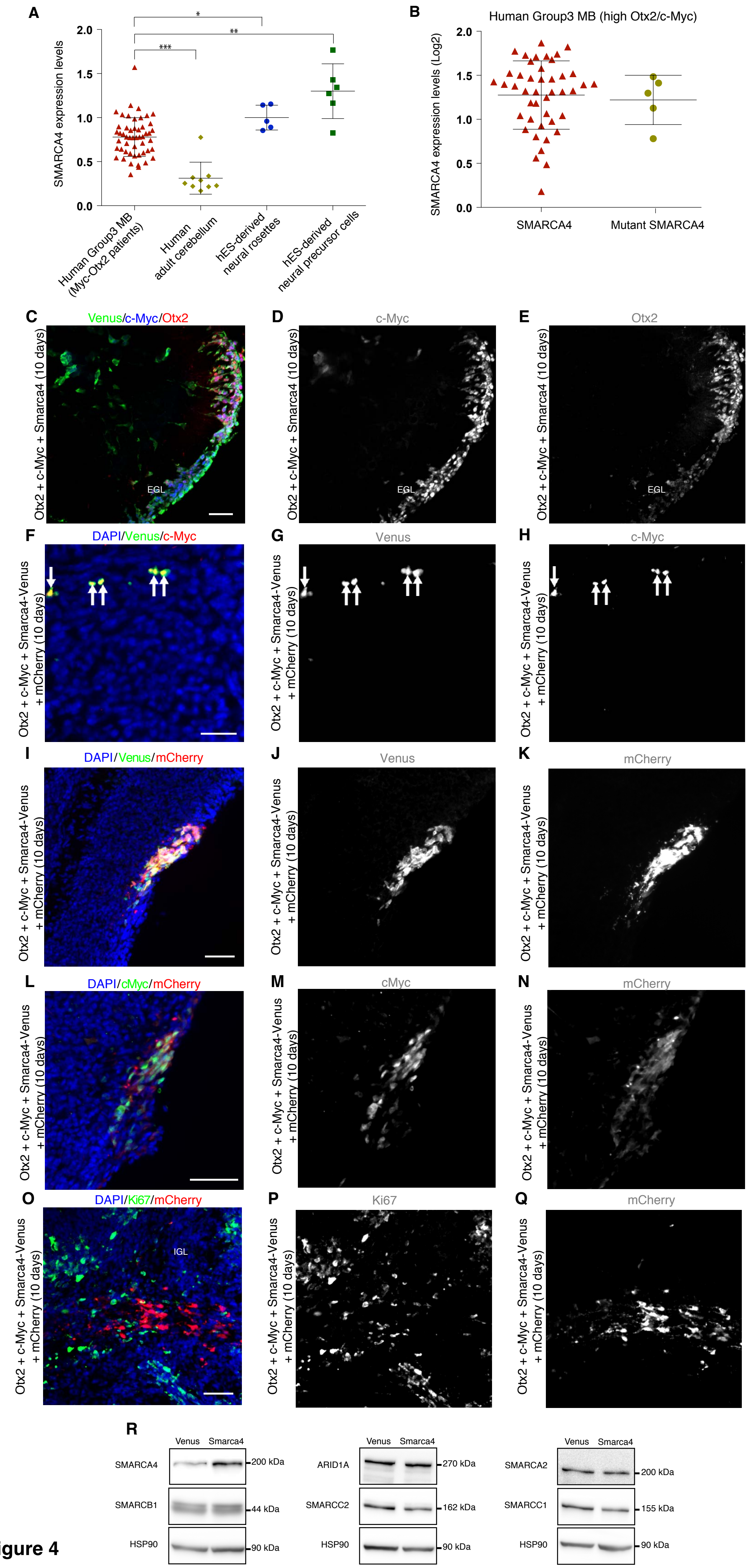


**Supplementary Figure 4**

**Supplementary Figure 4 | Smarca4 represses Otx2/c-Myc induced Group3 MB**

**(A)** SMARCA4 expression levels of human Group3 MB from patients with high levels of OTX2 and c-MYC expression, human adult cerebellum, ES-derived human neural rosettes and human ES-derived human neural precursors cells (https://hgserver1.amc.nl/cgi-bin/r2/main.cgi?&dscope=MB500&option=about_dscope#). Data were normalized to the average Smarca4 expression level in the GSE9921 dataset. **(B)** SMARCA4 (WT and Mutant) expression levels (Log2) of human Group3 MB from patients with high levels of OTX2 and c-MYC expression (https://hgserver1.amc.nl/cgi-bin/r2/main.cgi?&dscope=MB500&option=about_dscope#). Data were normalized to the average Smarca4 expression level in the GSE3526 dataset. (**C**-**E**) Confocal images of GFP (Venus) and c-Myc and Otx2 immunofluorescence of transfected cell clusters in CD1 mouse 10 days after transfection with pPBase + pPBMyc + pPBOtx2 + pPBSmarca4 + pPBVenus at P0. **(F-H)** Confocal images of GFP (SMARCA4-Venus), c-Myc immunofluorescence of transfected cell clusters in CD1 mouse 10 days after transfection with pPBase + pPBMyc + pPBOtx2 + pPBSmarca4-Venus + pPBmCherry at P0. **Arrows indicate double positive cells. (I-K)** Confocal images of GFP (SMARCA4-Venus) and mCherry immunofluorescence of transfected cell clusters in CD1 mouse 10 days after transfection with pPBase + pPBMyc + pPBOtx2 + pPBSmarca4-Venus + pPBmCherry at P0. **(L-N)** Confocal images of c-Myc and mCherry immunofluorescence of transfected cell clusters in CD1 mouse 10 days after transfection with pPBase + pPBMyc + pPBOtx2 + pPBSmarca4-Venus + pPBmCherry at P0. **(O-Q)** Confocal images of mCherry and ki67 immunofluorescence of transfected cell clusters in CD1 mouse 10 days after transfection with pPBase + pPBMyc + pPBOtx2 + pPBSmarca4-Venus + pPBmCherry at P0. (**R**) Western Blot analysis of human cerebellar progenitors (AF22 cells) 72h after electroporation with either pPBase + pPBVenus or pPBase + pPBSmarca4. Scale bars 100 µm (**C**). Scale bar 25 µm (**F**). Scale bar 50 µm (**I,L,O**). Unpaired t-test with Welch's correction two-tailed (**A**). *p value<0.05, **p value<0.01. ***p value<0.001.

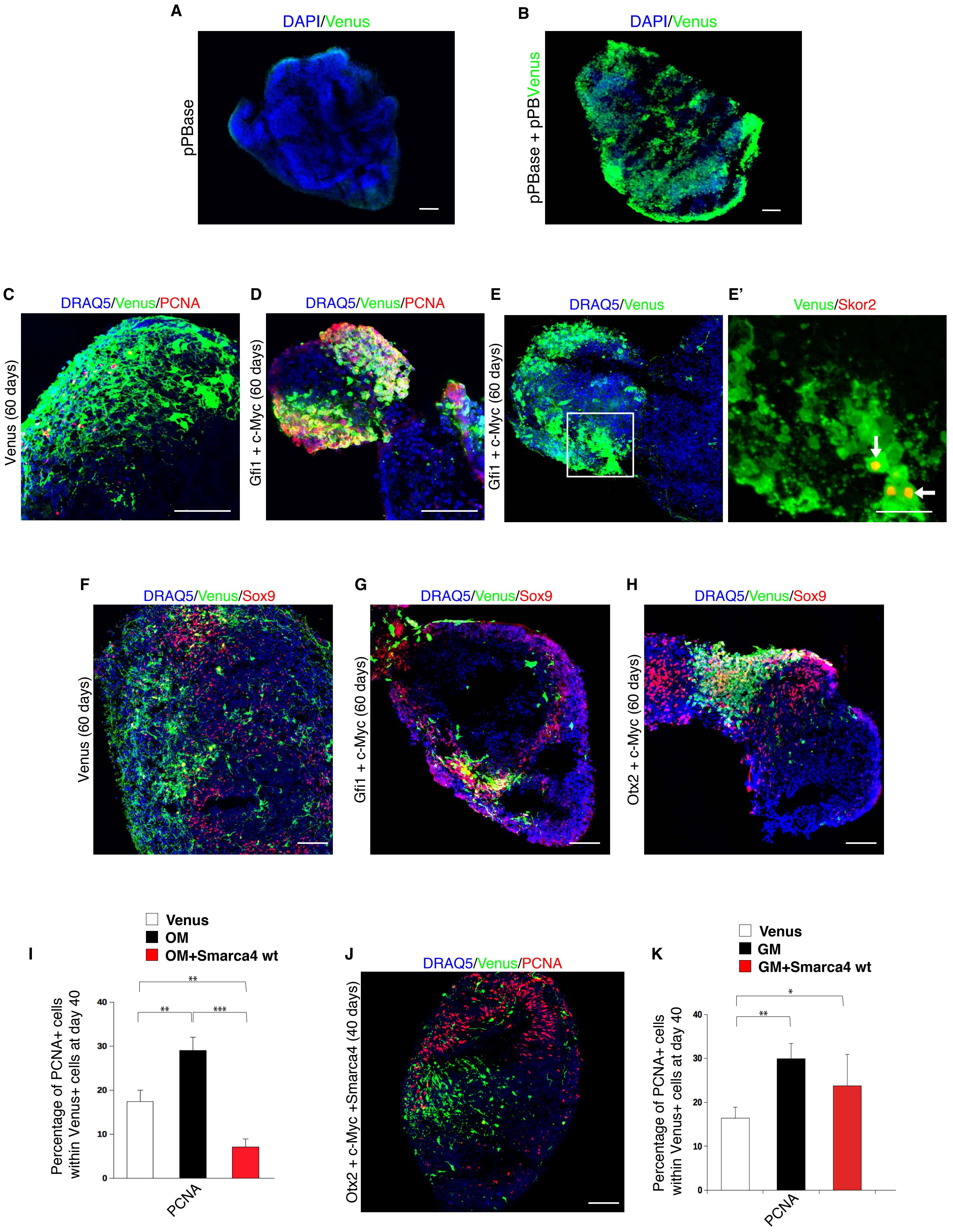


**Supplementary Figure 5**

**Supplementary Figure 5 | Cerebellar organoids electroporation with Gfi1/c-Myc and Otx2/c-Myc**

(**A,B**) Confocal images of DAPI staining and GFP (Venus) immunofluorescence of cerebellar organoids at day 60, electroporated with pPBase (**A**) and electroporated at day 35 with pPBase + pPBVenus (**B**). (**C**) Confocal images of GFP (Venus) and PCNA immunofluorescence of cerebellar organoids at day 60, electroporated at day 35 with pPBase + pPBVenus. (**D**) Confocal images of GFP (Venus) and PCNA immunofluorescence of cerebellar organoids at day 60, electroporated at day 35 with pPBase + pPBMyc + pPBGfi1 + pPBVenus. (**E**) Confocal images of GFP (Venus) and Skor2 immunofluorescence of cerebellar organoids at day 60, electroporated at day 35 with pPBase + pPBcMyc + pPBGfi1 + pPBVenus. Arrows point to double positive cells. The white square in (**E**) marks the region shown at higher magnification in (**E'**). (**F**) Confocal images of GFP (Venus) and Sox9 immunofluorescence of cerebellar organoids at day 60, electroporated at day 35 with pPBase + pPBVenus. (**G**) Confocal images of GFP (Venus) and Sox9 immunofluorescence of cerebellar organoids at day 60, electroporated at day 35 with pPBase + pPBMyc + pPBGfi1 + pPBVenus. (**H**) Confocal images of GFP (Venus) and Sox9 immunofluorescence of cerebellar organoids at day 60, electroporated at day 35 with pPBase + pPBMyc + pPBOtx2 + pPBVenus. (**I**) Quantification of cerebellar organoids GFP+/PCNA+ cells at day 40 electroporated at day 35 with either pPBase + pPBVenus (Venus) or pPBase + pPBMyc + pPBOtx2 + pPBVenus (OM) or pPBase + pPBMyc + pPBOtx2 + pPBVenus + pPBSmarca4 (OM+Smarca4 wt). (**J**) Confocal images of GFP (Venus) and PCNA immunofluorescence of cerebellar organoids at day 40, electroporated at day 35 with pPBase + pPBMyc + pPBOtx2 + pPBSmarca4. (**K**) Quantification of cerebellar organoids GFP+/PCNA+ cells at day 40 electroporated at day 35 with either pPBase + pPBVenus (Venus) or pPBase + pPBMyc + pPBGfi1 + pPBVenus (GM) or pPBase+pPBMyc+pPBGfi1+pPBVenus+pPBSmarca4 (GM+Smarca4 wt). Scale bars 250µm in (**A,B**), 100µm in (**C-H,J**), 40µm in (**E'**). Error bars in (**I,K**) represent standard error of the mean.Paired Student t-test, two tails. *p value<0.05, **p value<0.01. ***p value<0.001.

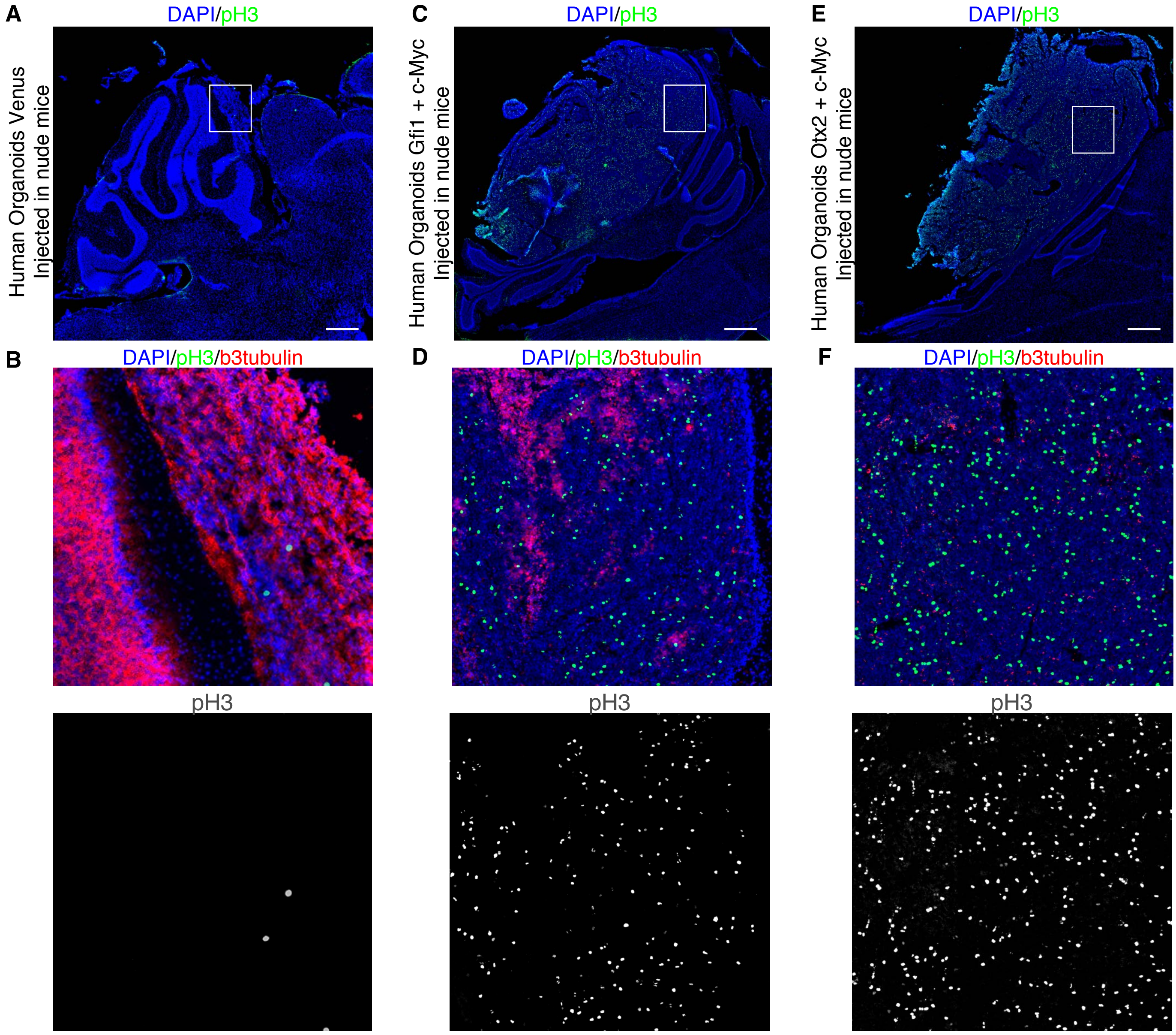


**Supplementary Figure 6**

**Supplementary Figure 6 | Cerebellar organoids electroporation with Gfi1/c-Myc or Otx2/c-Myc induces brain tumors**
(**A-C**) DAPI staining and pH3 immunofluorescence of sagittal brain sections of nude mice after injection of human cerebellar organoids electroporated with pPBase + pPBVenus (**A**, 72 days), pPBase + pPBMyc + pPBGfi1 + pPBVenus (**B,** 72 days), pPBase + pPBMyc + pPBOtx2 + pPBVenus (**C,** 30 days). The white squares in (**A,C,E**) mark the region shown at higher magnification in (**B,D,F**). Scale bars 1 mm in (**A,C,E**).

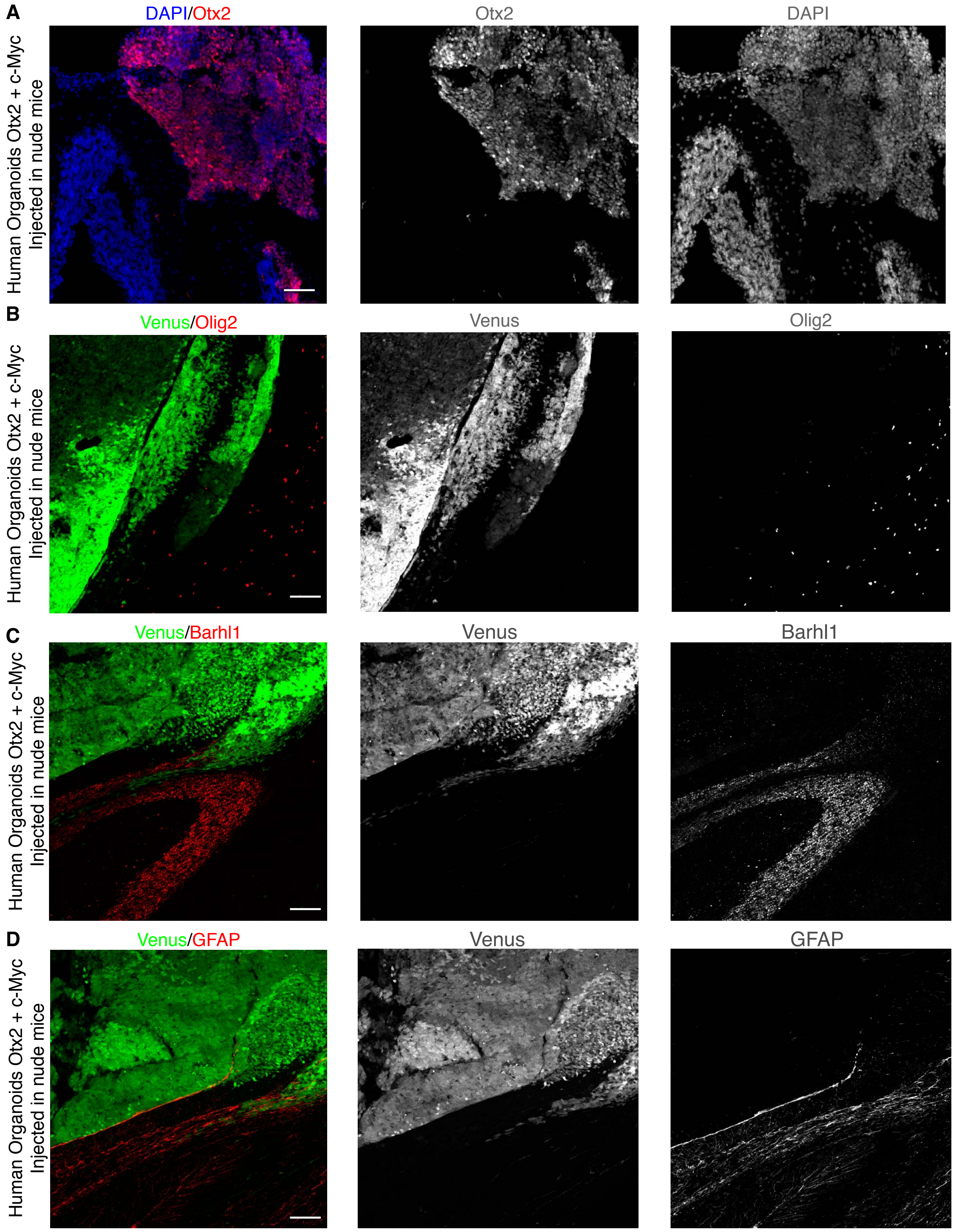


**Supplementary Figure 7**

**Supplementary Figure 7 | Cerebellar organoids electroporation with Otx2/c-Myc induces brain tumors *in-vivo***

(**A**) Confocal images of DAPI staining and Otx2 immunofluorescence of tumor in nude mouse 1 month after injection of human cerebellar organoids electroporated with pPBase + pPBMyc + pPBOtx2 + pPBVenus. (**B**) Confocal images of GFP (Venus) and Olig2 immunofluorescence of tumors in nude mouse 1 month after injection of human cerebellar organoids electroporated with pPBase + pPBMyc + pPBOtx2 + pPBVenus. (**C,D**) Confocal images of GFP (Venus), Barhl1 (**C**) and GFAP (**D**) immunofluorescence of tumors in nude mouse 1 month after injection of human cerebellar organoids electroporated with pPBase + pPBMyc + pPBOtx2 + pPBVenus. Scale bars 100 µm in (**A-D**).

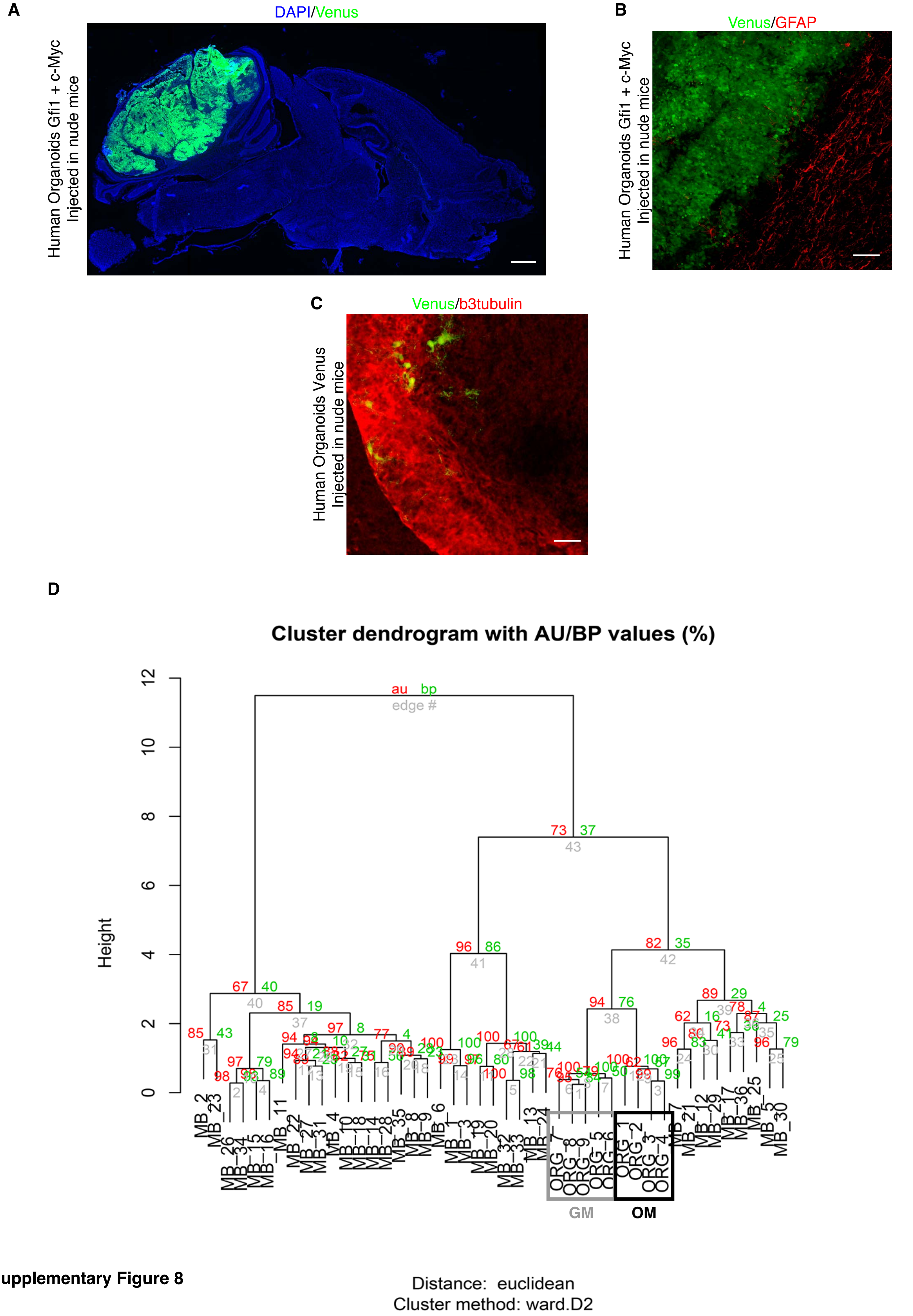


Supplementary Figure 8

**Supplementary Figure 8 | Cerebellar organoids electroporation with (either GM or OM induces brain tumors**

(**A**) DAPI staining and GFP (Venus) immunofluorescence of sagittal brain section of nude mouse after injection of human cerebellar organoids electroporated with pPBase + pPBMyc + pPBGfi1 + pPBVenus. (**B**) Confocal image of GFP (Venus) and GFAP immunofluorescence of tumor in nude mouse 72 days after injection of human cerebellar organoids electroporated with pPBase + pPBMyc + pPBGfi1 + pPBVenus. (**C**) b3tubulin and GFP (Venus) immunofluorescence of sagittal brain sections of nude mice after injection of human cerebellar organoids electroporated with pPBase + pPBVenus (72 days). **(D)** Cluster dendrogram with bootstrap analysis (1000 iterations) values on the 39 high-quality CpG islands in the Hovestadt set (Hovestadt et al. 2013) for the samples in the cohort. The bootstrap probability (BP) values are depicted in green, the approximately unbiased (AU) probability values (p-values) are reported in red. p-value of a cluster is a value between 0 and 1, which indicates how strongly the cluster is supported by data. Values are expressed in percentage. Edge numbers are in grey. OM Organoids (Org_OM, black); GM Organoids (Org_ GM, grey). Scale bars 1 mm in (**A**), 100 µm in (**B,C**).

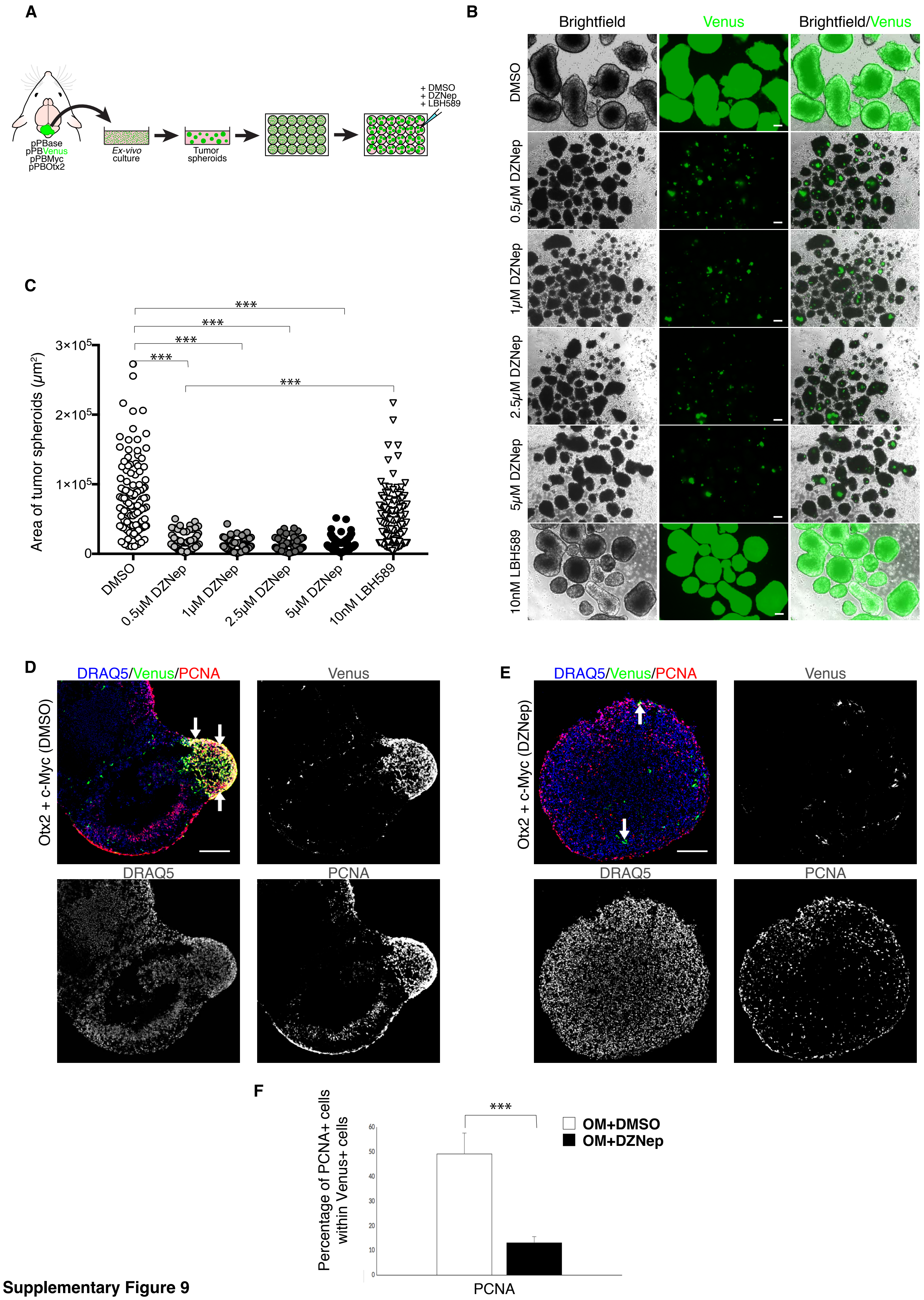


**Supplementary Figure 9**

**Supplementary Figure 9 | Histone methyltransferase inhibition reduces Otx2/c-Myc tumorigenesis**
(**A**) Schematic Representation of ex-vivo OM induced tumor culture and drug treatment. (**B**) Brightfield and Fluorescence images of OM induced tumor spheroids after 3 days of drug treatment. (**C**) Quantification of OM induced tumor spheroids area after 3 days of drug treatment. (**D-E**) Confocal images of GFP (Venus) and PCNA immunofluorescence of cerebellar organoids at day 57 electroporated at day 35 with pPBase + pPBMyc + pPBOtx2 + pPBVenus and treated with DMSO (**D**) and 5 µM DZNep (**E**) from day 37 to day 57. Arrows in (**D**) indicate Venus positive cells in an organized bud. Arrows in (**E**) indicate venus positive cells. (**F**) Quantification of cerebellar organoids GFP+/PCNA+ cells at day 57 electroporated at day 35 with pPBase + pPBMyc + pPBOtx2 + pPBVenus and treated with either DMSO or 5 µM DZNep. Error bars represent standard error of the mean. Paired Student t-test, two tails for organoids quantification. ***p value<0.001. Scale bars 100 µm in (**B,D,E**).

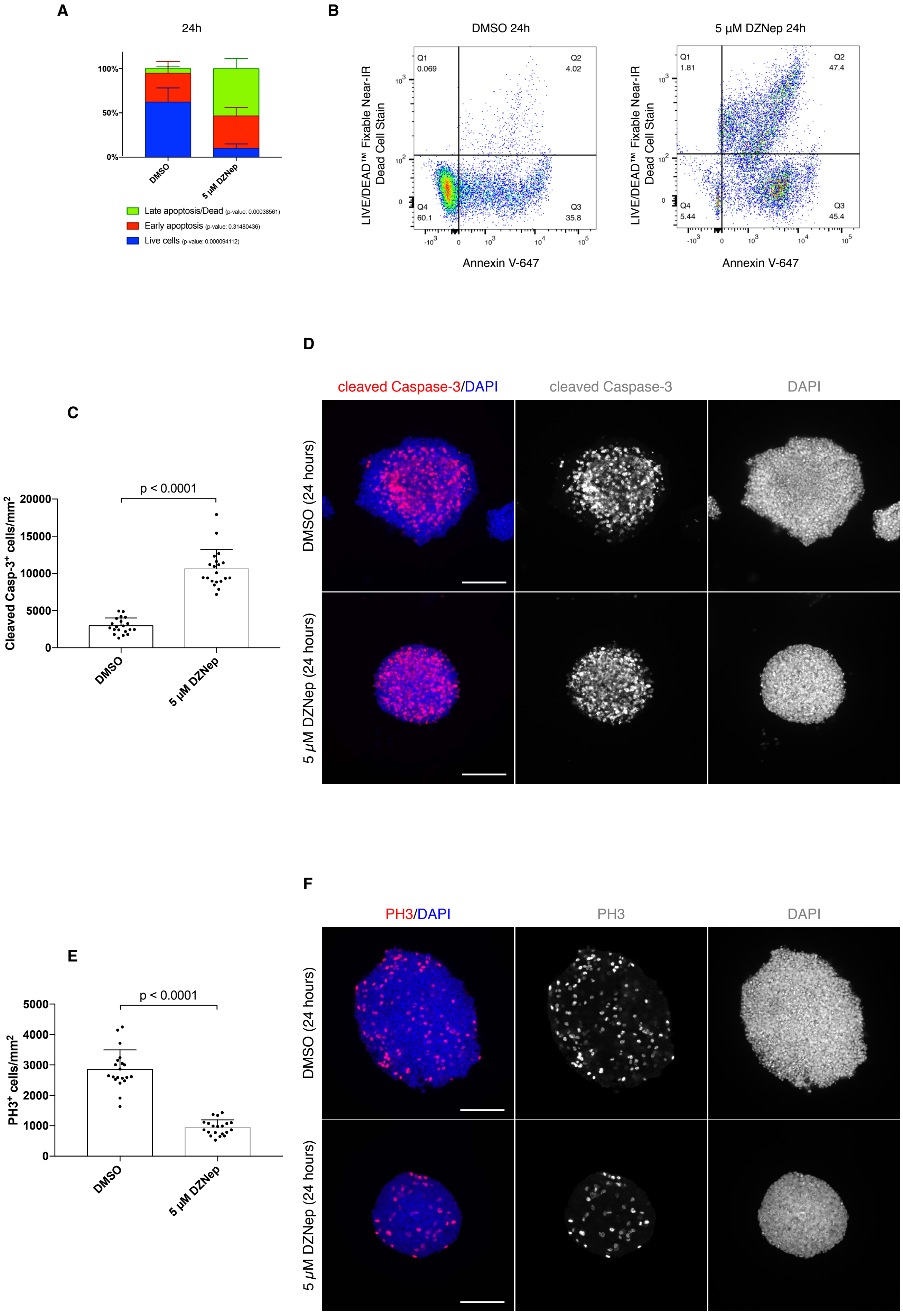


**Supplementary Figure 10**

**Supplementary Figure 10 | Histone methyltransferase inhibition increases cell death of OM-derived tumor spheroids**

(**A**) Histograms show FACS analysis of OM induced tumor spheroids cell death (late and early apoptosis) after 1 day of drug treatment (DMSO or DZNep). (**B**) Representative FACS analysis of OM induced tumor spheroids cell death (late and early apoptosis) after 1 day of drug treatment (DMSO or DZNep). (**C**) Quantification of OM induced tumor spheroids (staining with cleaved caspase-3) after 1 day of drug treatment (DMSO or DZNep). (**D**) Cleaved caspase-3 staining of OM induced tumor spheroids after 1 day of drug treatment (DMSO or DZNep). (**E**) Quantification of OM induced tumor spheroids (staining with PH3) after 1 day of drug treatment (DMSO or DZNep). Error bars in (**A,C,E**) represent standard deviation. (**F**) PH3 staining of OM induced tumor spheroids after 1 day of drug treatment (DMSO or DZNep). Scale bars 100 µm.
