# Supplementary material for: Modeling medulloblastoma in vivo and with human cerebellar organoids
Source: Nat Commun. 2020 Jan 29;11:583. doi: 10.1038/s41467-019-13989-3 (PMC6989674; doi:10.1038/s41467-019-13989-3)
Supplement: Supplementary file 6 — Reporting Summary [file 41467_2019_13989_MOESM6_ESM.pdf]

nature research

Corresponding author(s): Luca Tiberi

Last updated by author(s): Nov 27, 2019

Reporting Summary

Nature Research wishes to improve the reproducibility of the work that we publish. This form provides structure for consistency and transparency in reporting. For further information on Nature Research policies, see Authors & Referees and the Editorial Policy Checklist .

Statistics

For all statistical analyses, confirm that the following items are present in the figure legend, table legend, main text, or Methods section.

n/a	Confirmed	
☐	☒	The exact sample size (*n*) for each experimental group/condition, given as a discrete number and unit of measurement
☐	☒	A statement on whether measurements were taken from distinct samples or whether the same sample was measured repeatedly
☐	☒	The statistical test(s) used AND whether they are one- or two-sided<br>*Only common tests should be described solely by name; describe more complex techniques in the Methods section.*
☒	☐	A description of all covariates tested
☐	☒	A description of any assumptions or corrections, such as tests of normality and adjustment for multiple comparisons
☐	☒	A full description of the statistical parameters including central tendency (e.g. means) or other basic estimates (e.g. regression coefficient) AND variation (e.g. standard deviation) or associated estimates of uncertainty (e.g. confidence intervals)
☐	☒	For null hypothesis testing, the test statistic (e.g. *F*, *t*, *r*) with confidence intervals, effect sizes, degrees of freedom and *P* value noted<br>*Give P values as exact values whenever suitable.*
☒	☐	For Bayesian analysis, information on the choice of priors and Markov chain Monte Carlo settings
☒	☐	For hierarchical and complex designs, identification of the appropriate level for tests and full reporting of outcomes
☒	☐	Estimates of effect sizes (e.g. Cohen's *d*, Pearson's *r*), indicating how they were calculated

Our web collection on statistics for biologists contains articles on many of the points above.

Software and code

Policy information about availability of computer code

Data collection

Provide a description of all commercial, open source and custom code used to collect the data in this study, specifying the version used OR state that no software was used.

Data analysis

- DNA Methylation analysis: Generated methylation data were compared with the Heidelberg brain tumor classifier (Capper et al Nature 2018) (http://molecularneuropathology.org) to assign a subgroup score for the tumor compared to 91 different brain tumor entities. All tumors had a score of at least 0.8 in the reported methylation class.
EPIC BeadChip data were analyzed by means of R (V .3.4.3), using different packages: ChAMP pipeline (V.2.9.9, Morris et al 2014) for quality checks and filters, to calculate methylation levels and functionally annotate probes at the gene-level. Multidimensional scaling (MDS) on the cohort samples was performed using cmdscale function, with Euclidean distance. Heatmap depicting normalized beta values was created by means of pheatmap function, using Ward's minimum variance method (Murtagh et al. 2014) and Euclidean distance to cluster samples and probes. Low quality CpG islands among the 48 ones identified from Hovestadt and colleagues (Hovestadt et al. 2013) were removed from the analysis. Finally, bootstrap analyses were carried out using pvclust package (Suzuki and Shimodaira 2006).
- Gene expression analysis: All the sample sequence reads were mapped with STAR aligner (v2.5.3) using recommend parameters. To provide an estimate of gene expression and compute differential gene expression, the reads were proportionally assigned to the human gene transcripts (ENSEMBL HG38), based on the mappings using HT-SEQ count (http://www-huber.embl.de/users/anders/HTSeq. Differential gene expression analysis was performed using the gene raw counts, within the R/Bioconductor edgeR package. The differential gene expression pipeline within the edgeR package was customized to estimate the dispersion parameter for each library using the biological group dispersion and identify DE genes between treated versus the control samples. log2(fold-change) ≥ 1 and baseMean > 3 CPM were considered for differentially regulated genes and the P-value was adjusted for multiple testing using the Benjamini–Hochberg correction with a false discovery rate (FDR) ≤ 0.05. Differentially expressed gene lists obtained from low-level procedures were analysed for functional associations. Data were analysed through DAVID Bioinformatics Resources v6.8 using the suggested standard parameters.

- Kaplan-Meier analysis was performed using GraphPad Prism 7.0a

- Identification of genes differentially expressed: Genes that show differential expression (higher than 16 folds) compared to normal cerebellum have been identify using the online tool: https://hgserver1.amc.nl/cgi-bin/r2/main.cgi?&dscope=MB500&option=about_dscope#

For manuscripts utilizing custom algorithms or software that are central to the research but not yet described in published literature, software must be made available to editors/reviewers. We strongly encourage code deposition in a community repository (e.g. GitHub). See the Nature Research guidelines for submitting code & software for further information.

Data

Policy information about availability of data

All manuscripts must include a data availability statement. This statement should provide the following information, where applicable:

- Accession codes, unique identifiers, or web links for publicly available datasets
- A list of figures that have associated raw data
- A description of any restrictions on data availability

DNA Methylation Raw Data https://www.ncbi.nlm.nih.gov/geo/query/acc.cgi?acc=GSE128218

Field-specific reporting

Please select the one below that is the best fit for your research. If you are not sure, read the appropriate sections before making your selection.

[x] Life sciences [ ] Behavioural & social sciences [ ] Ecological, evolutionary & environmental sciences

For a reference copy of the document with all sections, see nature.com/documents/nr-reporting-summary-flat.pdf

Life sciences study design

All studies must disclose on these points even when the disclosure is negative.

Sample size

- All Kaplan-Meier survival curves present p values (statistical significance of the results was tested using the Log-rank test, Mantel-Cox).
- We obtained Medulloblastoma in 11/34 CD1 mice injected with Gfi1+c-Myc and 0/7 with only c-Myc and 0/11 with only Gfi1. Therefore, the animals analyzed are sufficient to conclude that the tumors are due to Gfi1+c-Myc overexpression.
- We obtained Medulloblastoma in 14/19 CD1 mice injected with Otx2+c-Myc and 0/7 with only c-Myc and 0/7 with Otx2. Therefore, the animals analyzed are sufficient to conclude that the tumors are due to Otx2+c-Myc overexpression.
- We obtained Medulloblastoma in 14/19 CD1 mice injected with Otx2+c-Myc and 0/9 with Otx2+C-Myc+Smarca4. Therefore, the animals analyzed are sufficient to conclude that Smarca4 is able to block Otx2+c-Myc tumorigenesis.
- We obtained Medulloblastoma in 5/5 nude mice injected with organoid modified with Otx2+c-Myc.
- We obtained Medulloblastoma in 6/6 nude mice injected with organoid modified with Gfi1+c-Myc.
- We obtained Medulloblastoma in 0/2 nude mice injected with organoid modified with Venus.

Data exclusions

No Data excluded

Replication

CD1 mice injected with Gfi1+c-Myc: 4 litters
CD1 mice injected with Otx2+c-Myc: 2 litters
CD1 mice injected with Otx2+c-Myc+Smarca4: 2 litters
CD1 mice injected with Otx2+c-Myc+Smarca4+Smarca4T910M: 2 litters
Nude mice injected with organoid modified with Otx2+c-Myc: 2 litters
Nude mice injected with organoid modified with Gfi1+c-Myc: 2 litters
Nude mice injected with organoid modified with Venus: 2 litters

Randomization

The CD1 and nude mice have been randomly selected before injection.

Blinding

Describe whether the investigators were blinded to group allocation during data collection and/or analysis. If blinding was not possible, describe why OR explain why blinding was not relevant to your study.

Reporting for specific materials, systems and methods

We require information from authors about some types of materials, experimental systems and methods used in many studies. Here, indicate whether each material, system or method listed is relevant to your study. If you are not sure if a list item applies to your research, read the appropriate section before selecting a response.

Materials & experimental systems

n/a	Involved in the study
☐	☒ Antibodies
☐	☒ Eukaryotic cell lines
☒	☐ Palaeontology
☐	☒ Animals and other organisms
☐	☒ Human research participants
☒	☐ Clinical data

Methods

n/a	Involved in the study
☒	☐ ChIP-seq
☒	☐ Flow cytometry
☒	☐ MRI-based neuroimaging

Antibodies

Antibodies used

All antibodies used are well described in Methods section.

Validation

Describe the validation of each primary antibody for the species and application, noting any validation statements on the manufacturer's website, relevant citations, antibody profiles in online databases, or data provided in the manuscript.

Eukaryotic cell lines

Policy information about cell lines

Cell line source(s)

human iPSC (ATCC-DYS0100) provided from ATCC.

Authentication

Describe the authentication procedures for each cell line used OR declare that none of the cell lines used were authenticated.

Mycoplasma contamination

The iPSC have been regularly tested for Mycoplasma contamination and resulted negative.

Commonly misidentified lines
(See ICLAC register)

Name any commonly misidentified cell lines used in the study and provide a rationale for their use.

Animals and other organisms

Policy information about studies involving animals; ARRIVE guidelines recommended for reporting animal research

Laboratory animals

We used CD1 mice (Charles River) and Foxn1nu mice (JAX#002019)

Wild animals

Provide details on animals observed in or captured in the field; report species, sex and age where possible. Describe how animals were caught and transported and what happened to captive animals after the study (if killed, explain why and describe method; if released, say where and when) OR state that the study did not involve wild animals.

Field-collected samples

For laboratory work with field-collected samples, describe all relevant parameters such as housing, maintenance, temperature, photoperiod and end-of-experiment protocol OR state that the study did not involve samples collected from the field.

Ethics oversight

This study was carried out in accordance with the recommendations of the Internal Review Board of University of Trento and approved by the Italian Ministry of Health.

Note that full information on the approval of the study protocol must also be provided in the manuscript.

Human research participants

Policy information about studies involving human research participants

Population characteristics

See Supplementary Table 2.

Recruitment

retrospective

Ethics oversight

This study was carried out in accordance with the recommendations of the Internal Review Board of the Bambino Gesù Ospedale Pediatrico (Rome, Italy) with written informed consent from all subjects. All subjects gave written informed consent in accordance with the Declaration of Helsinki. The protocol was approved by the Internal Review Board of the Bambino Gesù Ospedale Pediatrico (Protocol Number 1556_OPBG_2018).

Note that full information on the approval of the study protocol must also be provided in the manuscript.
