# Supplementary material for: Modeling medulloblastoma in vivo and with human cerebellar organoids
Source: Nat Commun. 2020 Jan 29;11:583. doi: 10.1038/s41467-019-13989-3 (PMC6989674; doi:10.1038/s41467-019-13989-3)
Supplement: Supplementary file 7 — Description of Additional Supplementary Files [file 41467_2019_13989_MOESM7_ESM.pdf]

Description of Additional Supplementary Files

Supplementary Data 1 Analysis of the differentially expressed genes (DEGs) in electroporated organoids compared to non-electroporated organoids. 26 genes are up-regulated in electroporated organoids; 7 genes are down-regulated in electroporated organoids; log fold change (lfc) > 1, Count per Million average expression (CPM) > 3, Benjamini–Hochberg adjusted p-value (FDR) ≤0.05.

Supplementary Data 2 Clinical and pathological features of the 36 primary MBs used for DNA methylation profiling. LC/A: large-cell anaplastic; SHH: Sonic Hedgehog; G3: Group3; G4: Group4.

Supplementary Data 3 Classification according to the brain tumor classifier (https://www.molecularneuropathology.org/mnp) of the DNA methylation profiles from our MB-like organoids.
